# Functions of ectodysplasin A2 receptor (EDA2R) in inducing capacitation of sperm in mice

**DOI:** 10.1007/s11626-025-01084-5

**Published:** 2025-07-21

**Authors:** Oluwakemi I. Anjorin, Takahiro Yamanaka, Masayuki Shimada

**Affiliations:** 1 Graduate School of Integrated Sciences for Life, Hiroshima University, 1-4-4 Kagamiyama, Higashihiroshima, Hiroshima 739-8528 Japan; 2 Graduate School of Innovation and Practice for Smart Society, Hiroshima University, 1-5-1 Kagamiyama, Higashihiroshima, Hiroshima 739-8529 Japan

**Keywords:** EDA-A2, EDA2R, Sperm capacitation, Mouse sperm motility, Cytokine

## Abstract

Sperm capacitation, a prerequisite for fertilization, is regulated not only by intrinsic signaling but also by paracrine factors within the female tract. Analysis of previously published RNA-seq datasets identified the ectodysplasin-A2 receptor (EDA2R), an X-linked member of the TNF-receptor superfamily, as a candidate regulator of this process. This study was conducted to test the hypothesis that the EDA-A2/EDA2R axis is a regulator that directly regulates sperm capacitation during fertilization process. Western blotting and immunofluorescence showed that EDA2R was localized in late spermatogenic cells and in the midpiece of epididymal sperm. Incubation of mouse sperm in HTF medium containing the corresponding ligand EDA-A2 (0–1 µg/mL) resulted in a dose-dependent improvement in the amplitude of lateral head displacement and curvilinear velocities. Ligand exposure promoted the appearance of capacitation hallmarks: tyrosine phosphorylation level was elevated within 30 min and the proportion of FITC-PNA positive, acrosome-reacted cells increased at 30 and 60 min (p < 0.05). The EDA-A2 treated sperm yielded a higher cleavage rate (78.5% vs. 48.3%) and a higher blastocyst formation rate (97.6% vs. 88.4%) after in vitro fertilization. qPCR in hormonally synchronized females revealed transient ovarian and prolonged oviductal *Eda-a2* upregulation surrounding ovulation, suggesting that the ligand is present at the site of sperm-oocytes fertilization. These results clarify that EDA-A2/EDA2R is a rapid physiological driver of sperm capacitation. This provides a tractable cytokine axis for optimizing assisted reproduction.

**Supplementary Information:**

The online version contains supplementary material available at 10.1007/s11626-025-01084-5.

## Introduction

Sperm capacitation is a prerequisite for successful fertilization, involving a series of biochemical and physiological changes within sperm after ejaculation. The mechanism of inducing capacitation in sperm has been reported in detail in molecular level (Gervasi and Visconti 2016; Yanagimachi 2022). The increasing level of progesterone after ovulation might be involved in the induction of sperm capacitation during the fertilization process (Vanderhyden and Tonary 1995; Romarowski *et al*. 2016). However, there are many unclear factors that induce capacitation from the outside of sperm. In our previous study (Shimada *et al*. 2008), it has been reported that hyaluronan fragments, generated by sperm-secreted hyaluronidase, can stimulate cytokine and chemokine production in cumulus cells during fertilization process, potentially enhancing sperm capacitation. These findings suggested that immune signaling might play functional roles in the fertilization process.

RNA-seq database (DRA007935) of our previous study (Umehara *et al*. 2019) revealed the expression of one cytokine receptor family, ectodysplasin A2 receptor (EDA2R) in mouse sperm. EDA2R is a transmembrane receptor belonging to the tumor necrosis factor (TNF) receptor superfamily and specifically binds to its ligand EDA-A2 (Yan *et al*. 2000). Ligand engagement activates intracellular signaling cascades such as the NF-κB and JNK pathways, which regulate mitochondrial function, cell survival and apoptosis (Yan *et al*. 2000; Verhelst *et al*. 2015). Although EDA2R is encoded by *Eda2r* localizing on the X chromosome and has been extensively studied in somatic tissues (Xing *et al*. 2023), its physiological role in male germ cells, including sperm, remains largely unknown.

This study aims to investigate whether the EDA-A2–EDA2R signaling pathway contributes to the regulation of sperm capacitation. We hypothesized that EDA-A2 might modulate capacitation-associated changes in sperm function through EDA2R-mediated signaling. To test this hypothesis, we investigated the effects of EDA-A2 on sperm motility and function including capacitation markers, and *Eda-a2* expression in female reproductive tissue. Clarifying whether the EDA-A2/EDA2R pathway is involved in sperm function may contribute to the understanding of a new regulatory mechanism of sperm maturation and capacitation.

## Materials & methods

### Chemicals and animals

Unless otherwise noted, chemicals used in this study were purchased from Sigma-Aldrich St. louis, MO or Nacalai tesque Kyoto, Japan. Equine chorionic gonadotropin (eCG) and human chorionic gonadotropin (hCG) were purchased from Asuka Seiyaku.

C57BL/6NJ male mice (8–12 weeks old) and Crl: CD1 (ICR) female mice (3 wk old) were purchased from Jackson Laboratory Japan Yokohama-shi, Japan. Animals were housed in an environmentally controlled room with a 12-h light/dark cycle, a temperature of 23 ±  3 °C, and free access to laboratory food (MF; Oriental Yeast Co., Ltd Tokyo, Japan.) and tap water. All animal experimental procedures were reviewed and approved by the Animal Care and Use Committee of Hiroshima University (C23-36) and conducted according to regulations.

### Western blotting

Protein samples of sperm collected from the cauda epididymis, testis, and liver as a positive control were obtained using cell lysis buffer (04,719,964,001, cOmplete™ Lysis-M EDTA-free, Roche Basel, Switzerland). For analyzing tyrosine phosphorylation, sperm was collected in human tubal fluid (HTF) medium (Composition is listed in the Supplemental Table 1), incubated for 0, 30 min or 1 h, and then lysed. The extracts were separated by SDS–polyacrylamide gel (10%) electrophoresis and transferred to polyvinylidene fluoride membranes (10,600,069, Cytiva Marlborough, MA), as described in our previous study (Yamanaka *et al*. 2025). Membranes were blocked with 5% (w/v) BSA in Tris-buffered saline and Tween 20 (TBST), incubated overnight at 4 °C with primary antibodies [EDA2R, 1:1000, bs-7111R, Bioss Antibodies; phosphotyrosine antibody 1:10,000, 9411S, Cell Signaling; α/β-Tubulin, 1:1000, 2148S, Cell Signaling Danvers, MA]. The membranes were washed with TBST and incubated with HRP-labeled anti-rabbit IgG antibody (1:4000; 7074S; Cell Signaling). The bands were visualized using an Enhanced Chemiluminescence detection system (RPN2232, Cytiva) and ChemiDocTM MP Imaging System (Bio-Rad Laboratories Hercules, CA).

### Immunofluorescence

Testis was fixed overnight in 4% (w/v) paraformaldehyde/phosphate-buffered saline (PBS), dehydrated in 70% (v/v) ethanol, and embedded in paraffin. Sperm was mounted on glass slides, air-dried, fixed with 4% paraformaldehyde for 30 min, and permeabilized with 0.3% (v/v) Triton X-100 in PBS for 30 min at room temperature. The Sects. (4 μm) and slides were blocked with goat serum and incubated overnight with primary antibodies [rabbit anti-EDA2R, 1:100 and mouse anti-SP56, 1:100, 55,101, QED Biologicals]. After washing, Cy3-labeled goat anti-rabbit IgG (1:200, C-2306, Sigma), FITC-labeled goat anti-mouse IgG and DAPI (VECTESHIELD Mounting Medium with DAPI, H-1200, Vector Laboratories Newark, CA) were used to visualize the antigens and nuclei. Digital images were obtained using an APX100 Digital Imaging System and CellSens imaging software (EVIDENT), as described in our previous study (Younus *et al*. 2024).

### Assessment of sperm motility

Sperm motility was evaluated using computer-assisted sperm analysis (CASA) as described in our previous study (Umehara *et al*. 2019). Cauda epididymis sperm recovered from male mouse were dispersed in 500 µL of HTF, and incubated at 37 °C and 5% CO_2_ for 1 h in HTF containing EDA-A2 (the specific ligand of EDA2R receptor; 922-ED, R&D Systems Minneapolis, MA) at different concentrations (0,1,10,100 and 1,000 ng/mL). Thereafter, sperm tracks were captured at 60 Hz using the CASA system (HT CASA-Ceros II; Hamilton Thorne Beverly, MA) for 0.5 s (45 frames). More than 100 individual trajectories were recorded. The following sperm motility parameters were analyzed: motile sperm were denoted by an average path velocity of > 10 μm/s and a straight-line velocity of > 0 μm/s.

### Evaluation of acrosome reaction

The acrosome status was assessed using 25 µg/mL fluorescein isothiocyanate-conjugated peanut agglutinin (FITC-PNA; L7381, Sigma Aldrich) and 12 µM propidium iodide (PI; L7011, Invitrogen  Carlsbad, CA), as described in our previous study (Yamanaka *et al*. 2025). After incubation in the dark at 37 °C for 10 min. The fluorescence of FITC and PI were analyzed by flow cytometry (Attune™ CytPix Flow Cytometer, Thermo Fisher Scientific Inc.) using 530/30 and 695/40 nm bandwidth filters, respectively. Acrosome reacted sperm was analyzed—FITC fluorescence positive, but PI negative gate (Q4). A total of 100,000 sperm events were analyzed. The sperm population is shown in Supplementary Fig. 1.

### In vitro fertilization (IVF)

As detailed in our earlier study (Yamanaka *et al*. 2025), IVF was performed. Three weeks old C57BL/6NJ female mice were injected intraperitoneally with 4 IU eCG to stimulate follicle development and 5 IU hCG at 48 h later. Cumulus-oocyte complexes (COC) collected from the oviductal ampulla 16 h after hCG injection were placed into 100 μL of HTF medium. Sperm were collected from the cauda epididymis of 15 wk or older C57BL/6N male mice in 100 μL of HTF medium. After 60 min of incubation with or without EDA-A2 (1000 ng/mL), the sperm was added into HTF medium at a final concentration of 2 × 10^5^ sperm/mL, and co-incubated with the up to 15 COCs (ranging from 10 to 15) per culture drop. After 6-h incubation at 37 °C and 5% CO_2_, oocytes were washed three times thoroughly and cultured in KSOM medium (MR 106-D; Sigma). The cleavage rate was determined on Day 1 (Day 0 = day of IVF), and the appearance of blastocysts was recorded on Day 5.

### qPCR

Immature female mice were administered 4 IU of eCG for 48 h, followed by an injection of 5 IU of hCG. Ovaries and oviducts were collected from immature mice that had not administered hormones, 48 h after eCG administration (= 0 h before hCG administration), and 4, 8, and 12 h after hCG administration. Granulosa cells were isolated from the antral follicles by needle puncture in Dulbecco's Modified Eagle Medium and recovered from the medium.

Total RNA was extracted from ovaries, oviducts, and granulosa cells using the RNeasy Mini Kit (74,106, QIAGEN), as described in our previous study (Yamanaka *et al*. 2025). The cDNA was synthesized from 200 ng (10 ng/µL) of total RNA using oligo(dT)_15_ primers (3805, Takara Bio Inc., Shiga, Japan) and an Avian myeloblastosis virus (AMV) reverse transcriptase (M5101, Promega Madison, WI). The cDNA and primers were added to Power SYBR Green PCR Master Mix (4,367,659, Applied Biosystems, Foster City, CA,) in a total reaction volume of 15 μl. Conditions were set to the following parameters: 10 min at 95 °C followed by 50 cycles each of 15 s at 95 °C and 1 min at 60°C. Cycles were repeated 45 times. The following primers were used: *Eda-a2*, 5′-CTGGTGCTGCTGATAAAACTGG-3’ (forward) and 5′- TTGGCAAACAGCTGTGAGGA-3’ (reverse); *Rpl19*, 5′- GGCATAGGGAAGAGGAAGG-3’ (forward) and 5′- GGATGTGCTCCATGAGGATGC-3’ (reverse). Expression was first normalized to housekeeping gene *Rpl19*, and fold change was calculated relative to the mean of the control samples.

### Statistical analysis

Quantitative data were presented as means ± SEM. Student's t-test was used to compare the two groups. Differences between groups were assessed using one-way analysis of variance (ANOVA). When ANOVA was significant, differences among values were analyzed using Tukey's Honest Significant Difference test for multiple comparisons. Cleavage and blastocyst rates were analyzed using chi-squared test. R (version 4.3.1) was used for statistical analysis. Statistical value of p < 0.05 was defined as a significant difference.

## Results

### EDA2R is expressed late spermatogenic cells and the midpiece of epididymal sperm

**Figure 1.**
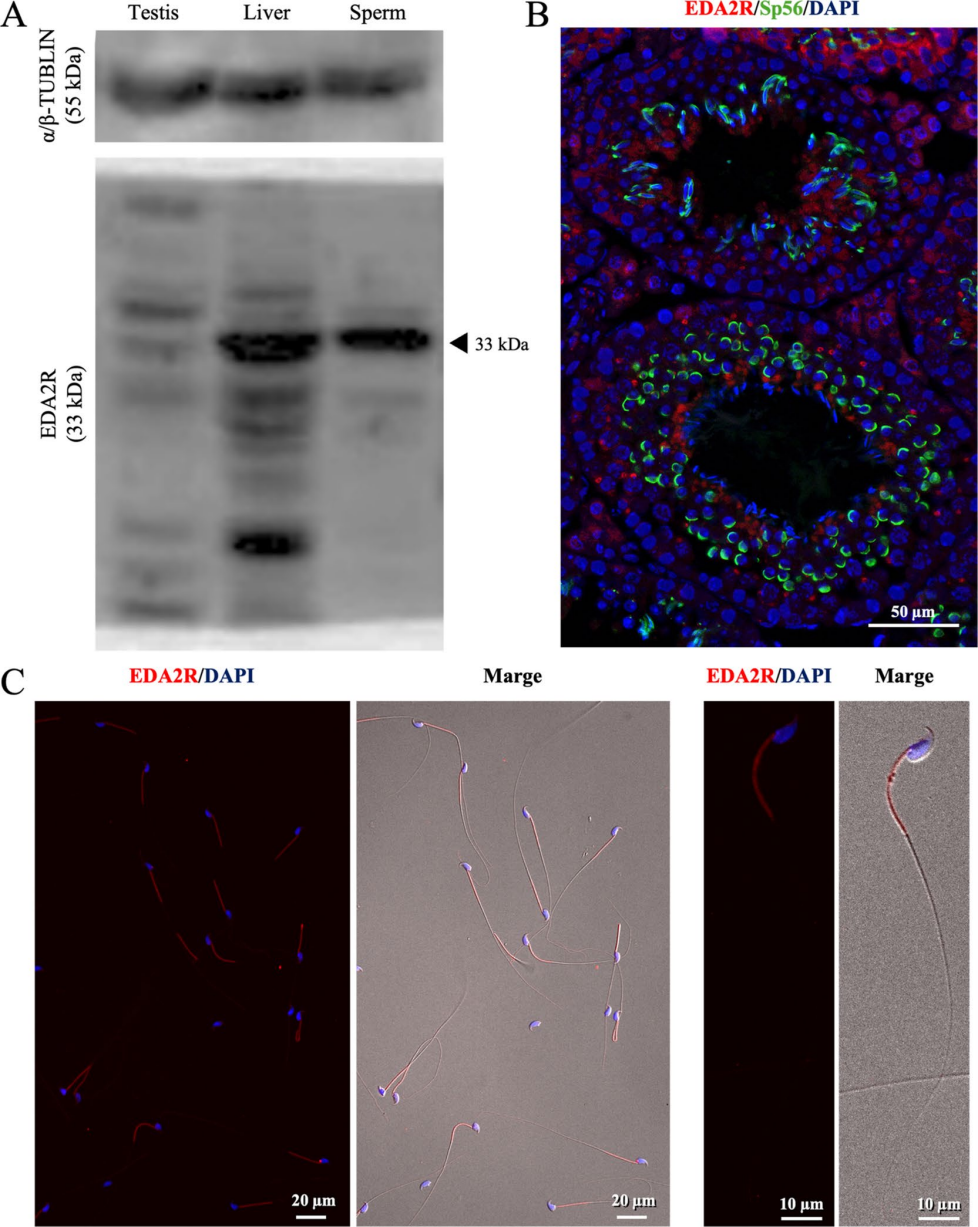
Expression profile and localization analysis of EDA2R in testes and sperm. (***A***) Expression of EDA2R in mouse epididymal sperm and testis. α/β-tubulin was used as a loading control. Lysates prepared from the liver were used as a positive control. (***B***) Localization of EDA2R in the testis. Cross-sections of mouse testes were stained with antibodies to visualize EDA2R (*red*) or Sp56 (*green*), which is a marker of the acrosome in round spermatids and sperm at 12 wk. (***C***) Representative images of the epididymal sperm fluorescence signal with EDA2R.

When EDA2R protein expression was analyzed by Western blotting, the positive band of EDA2R was detected in the epididymal sperm as like that in the liver used as a positive control (Fig. 1*A*). However, the intensity of positive signal was much weaker in the whole testis (Fig. 1*A*). In immunofluorescence study using mouse testis, EDA2R-positive signals were observed in approximately half of the cells double-stained with zona pellucida 3 receptor (ZP3R; formerly known as Sp56), a marker for spermatogenic cells after the round spermatid stage Fig. 1*B*). The EDA2R-positive signals were observed more frequently as the cells approached the lumen, though EDA2R was also expressed in part of the Leydig cells and Sertoli cells (Fig. 1*B*). In epididymal sperm, EDA2R was expressed in the midpiece of all sperm, and weak positive signals were also observed in the head (Fig. 1*C*).

### EDA2R Activation shifts sperm toward a high-velocity, high-amplitude subpopulation

**Figure 2.**
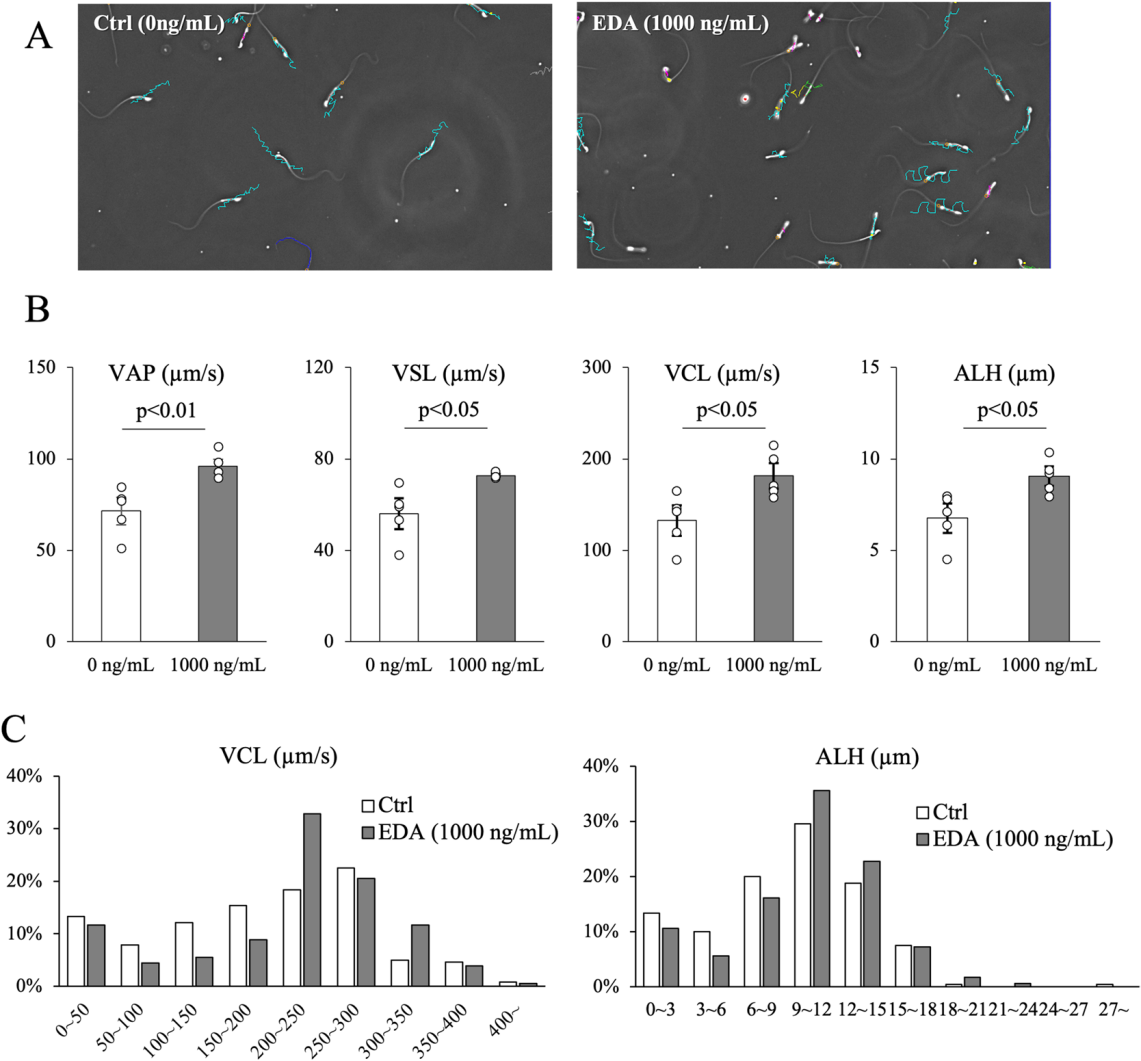
Changes in sperm kinetics induced by EDA-A2 stimulation. (***A***) Tracks of sperm incubated with EDA-A2 (EDA) or without (Ctrl; 0 ng/mL) were determined using the CASA system. (***B***) Velocity parameters of epididymal sperm incubated with 1000 ng/mL EDA-A2 ligand for 60 min. VAP; average path velocity, VSL; Straight-Line Velocity, VCL; Curvi-Linear Velocity, ALH; amplitude of lateral head displacement. (***C***) The histograms representation of the VCL and ALH of mouse sperm incubated with EDA or without (Ctrl). Values obtained from more than five replications (p < 0.05 was considered statistically significant; Student's t-test).

EDA2R was expressed in the midpiece of sperm; therefore, epididymal sperm was incubated with EDA-A2, a specific ligand of EDA2R. To investigate the effect of EDA-A2 binding to EDA2R on sperm motility, different concentrations of EDA-A2 were added to HTF medium, and suspensions of epididymal sperm were incubated for 60 min. It was observed that curvilinear velocity (VCL) and amplitude of lateral head displacement (ALH) increased in a dose-dependent manner, reaching their maximum values at 1,000 ng/mL (Supplementary Fig. 2). When directly compared with 1,000 ng/mL of EDA-A2, significant increases were observed in all parameters including the average path velocity (VAP) and straight-line velocity (VSL; Fig. 2*A*, *B*).

Conventional statistical analyses of kinetic parameters obtained with a CASA system rely on the mean values of hundreds of sperm tracks. Because these means do not reflect the variability inherent in a sperm sample, individual motility data were plotted as histograms data to verify the existence of heterogeneous motility subpopulations that display distinct kinematic patterns. Only “motile sperm” with VCL > 20 µm/s were analyzed, and high-range sperm were defined as those with VCL > 200 µm/s and ALH ≥ 9 µm. The addition of EDA-A2 resulted in a similar trend to the analysis of average data, with VCL and ALH shifted toward higher values. Furthermore, High VCL accounted for 51% in the control group and 69% in the EDA-A2 group. High ALH accounted for 57% in the control group, while 74% were in the EDA-A2 group (Fig. 2*C*).

### Ligand-activated EDA2R drives early tyrosine phosphorylation and acrosome reaction

**Figure 3.**
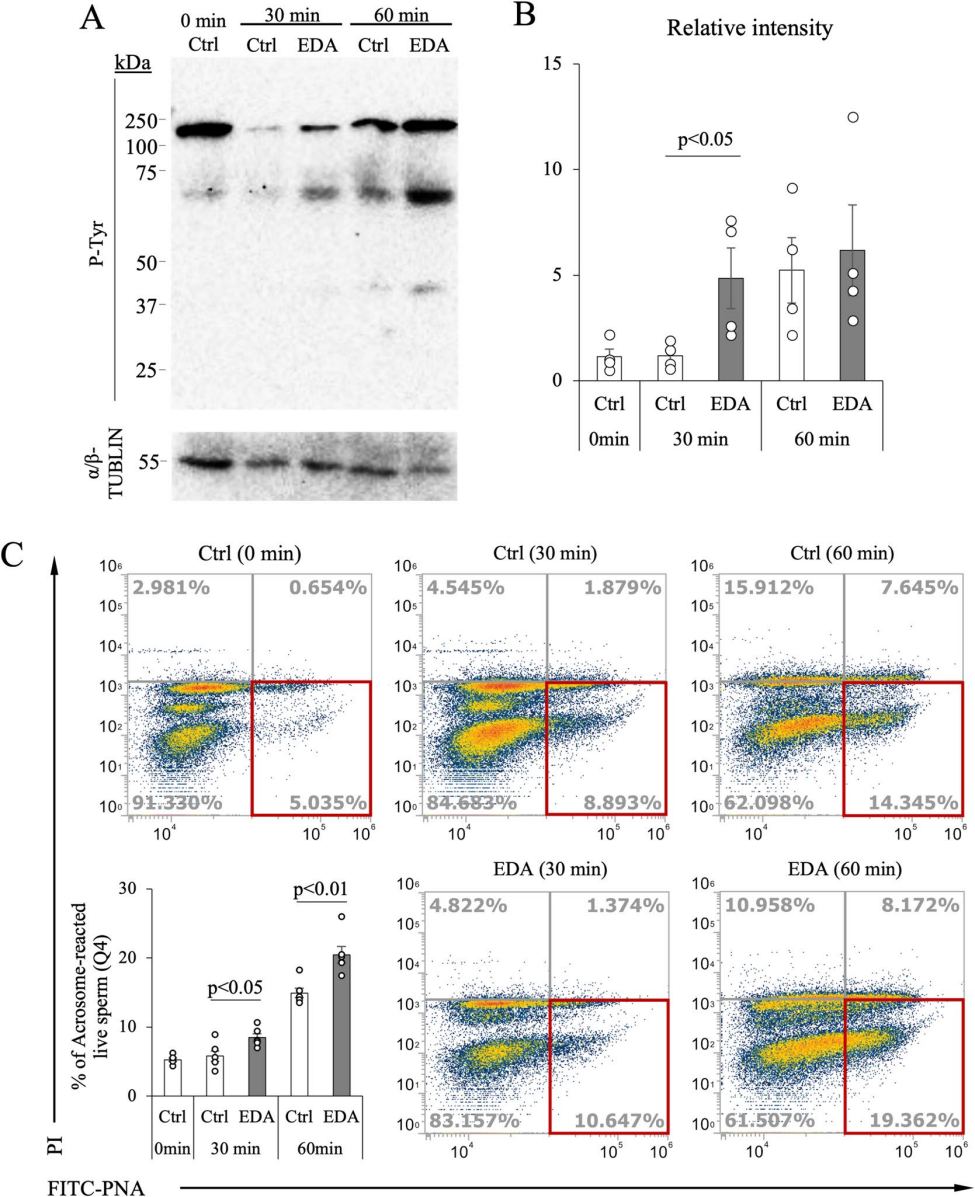
Time-dependent tyrosine phosphorylation and induction of the acrosome reaction. (***A***) Western blot analysis for phosphotyrosine (P-Tyr) in non-cultured (0 min) and cultured (30 and 60 min) sperm in HTF with or without EDA-A2 (EDA; 1000 ng/mL). (***B***) A relative quantitative analysis of P-Tyr and α/β-TUBLIN was performed using Western blotting. (***C***) Flow cytometry analysis was performed to distinguish between sperm cells that underwent an acrosome reaction using fluorescent isothiocyanate-labeled peanut agglutinin (PNA-FITC) and those that did not. Dead cells were distinguished from live cells using propidium iodide (PI). The percentage of acrosome-reacted live sperm (PI-, PNA-FITC +) was evaluated by flow cytometry in the fourth quadrant (4Q; red square). Values obtained from more than four replications (p < 0.05 was considered statistically significant; Student's t-test).

Capacitation is a biochemical process that sperm undergo after entering the female reproductive tract following ejaculation, and it is considered a prerequisite for the acrosome reaction. Therefore, the tyrosine phosphorylation, an indicator of capacitation was examined in sperm incubated with or without EDA-A2. When cultured in HTF medium for 30 min, the addition of EDA-A2 (1000 ng/mL) significantly increased the intensity of the tyrosine phosphorylation signals as compared with in sperm incubated without EDA-A2 (control). After 60 min of incubation, the intensity of tyrosine phosphorylation signals was increased in control to the same level to EDA-A2 group (Fig. 3*A*, *B*).

Since only sperm that undergoes acrosome reaction can fertilize to the matured oocyte, acrosome reaction in vitro was analyzed using a FITC-PNA staining method. Consistent with the tyrosine phosphorylation levels, sperm treated with EDA-A2 initiated acrosome reaction earlier and showed a significantly higher proportion of sperm that underwent acrosome reaction at both 30 and 60 min of incubation (Fig. 3*C*).

To confirm the effect of EDA-A2 on sperm fertilization potential, in vitro fertilization (IVF) was performed. Sperm was incubated in HTF media with or without EDA-A2 1000 ng/mL for 60 min. Thereafter, the sperm cells were used for in vitro fertilization. As a result, EDA-A2 significantly improved the cleavage rate and subsequent blastocyst formation rate compared to the control group (Table 1).

**Table 1.** Results of in vitro fertilization performed with sperm preincubated with EDA-A2 (1000 ng/mL) for 60 min

	No. (%) of oocytes		No. (%) of blastocysts
	MII	Cleaved (/MII)	(/cleaved)
Ctrl	89	43 (48.3%) ^a^	38 (88.4%) ^c^
EDA-A2	107	84 (78.5%) ^b^	82 (97.6%) ^d^

^a,b; c,d^; Different *alphabetic symbols* indicate significant differences (p < 0.05 was considered statistically significant; chi-squared test)

The values were obtained from six replications

### Transient ovarian and prolonged oviductal elevation of Eda-a2 around ovulation

**Figure 4.**
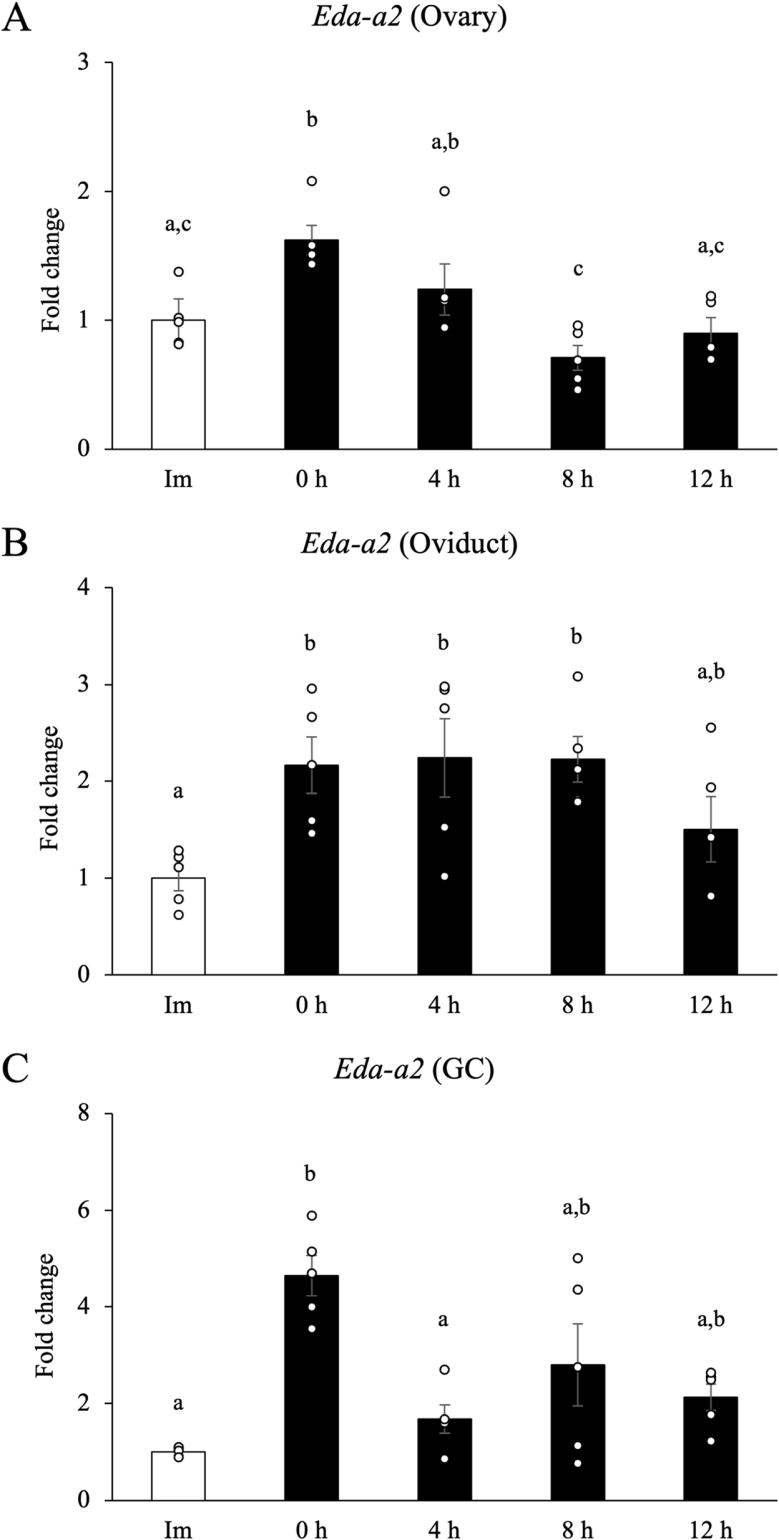
Gene expression dynamics of *Eda-a2* in the ovaries, oviducts, and granulosa cells in response to eCG/hCG Stimulation. (***A-C***) Samples were collected from immature mice that had not administered hormones, 48 h after eCG administration (= 0 h before hCG administration), and 4, 8, and 12 h after hCG administration. The expression levels of *Eda-a2* gene were normalized according to that of *Rpl19*. For reference, the Immature (Im) value was set as 1, and the data are presented as fold change. GC; granulosa cells. Values obtained from more than four replications. Different *letters* represent significantly different groups (p < 0.05 was considered statistically significant; Tukey's Honest Significant Difference test).

To evaluate the presence and physiological functionality of EDA-A2 in vivo, the gene expression levels of *Eda-a2* in the ovaries, granulosa cells and oviducts were examined at the following time points: immature (Im), 48 h after eCG administration (0 h), 4 h, 8 h and 12 h after hCG administration. Gene expression was consistent with follicular development, with the highest expression observed 48 h after eCG administration in all samples. In the ovaries and granulosa cells, expression gradually decreased over time following hCG administration (Fig. 4*A*, *B*). In contrast, in the oviducts, the increased expression was maintained until 8 h after hCG administration (Fig. 4*C*).

## Discussion

The EDA-A2/EDA2R axis constituted a previously unrecognized, rapid-acting regulator of sperm capacitation and fertilizing ability. Exposure to EDA-A2 promoted all typical characteristics of capacitation (hyperactivated-like motility; high VCL & ALH, rapid tyrosine phosphorylation, and early acrosome reaction) and improved fertilization rates and blastocyst formation rates in vitro. The total tyrosine phosphorylation levels of EDA-A2-treated sperm and control sperm converged after 60 min, but the two groups still showed differences in hyperactivated motility (Fig. 2*B*, *C* and Fig. 3*A*). Changes in sperm motility during capacitation are induced by an increase in calcium ions and the phosphorylation of tyrosine residues on specific proteins. EDA-A2 treatment was able to activate the signaling pathways involved in sperm capacitation early. However, this induction is not governed by a single pathway, which suggests that capacitation was delayed but eventually induced in the control as well. For instance, in our experimental IVM medium, we added factors known to induce capacitation, such as albumin and bicarbonate. While the results from this study alone cannot clarify whether EDA-A2 interacts with these factors or induces.

EDA2R is an X-linked member of the TNF-receptor (TNFR) superfamily whose canonical signaling engages TRAF6–NF-κB and JNK cascades (Yan *et al*. 2000; Sinha *et al*. 2002; Verhelst *et al*. 2015; Xing *et al*. 2023). In somatic epithelium, these pathways modulate mitochondrial metabolism and redox tone (Laforge *et al*. 2016; Carrà *et al*. 2022). EDA2R is believed to function as a ligand-dependent receptor. Unlike TNFR1/TNFR2, which can undergo ROS-driven ligand-independent clustering and signaling, direct autonomous activation of EDA2R under stress has not been documented. The midpiece localization of EDA2R therefore provides a mechanistic route by which EDA-A2 may boost mitochondrial ATP production, translating into higher flagellar beat amplitude and velocity. The early wave of protein-tyrosine phosphorylation parallels the cAMP/PKA pathway that triggers capacitation; NF-κB can feed into this pathway through PI3K and soluble adenylyl-cyclase, offering a plausible molecular bridge to induce sperm capacitation (Visconti *et al*. 1995; Jin and Yang 2016; Tsirulnikov *et al*. 2019; Li *et al*. 2023; Takei. 2024; Vahedi Raad et al 2024). Taken together, our findings position EDA-A2/EDA2R as an upstream trigger of capacitation-related signaling; however, the precise downstream cascade linking receptor engagement to cAMP/PKA activation and mitochondrial remodeling in sperm remains to be elucidated.

The ovarian/oviductal *Eda-a2* expression profile reinforces the idea that EDA-A2 acts as a paracrine “fertilization timer”. FSH-dependent follicular growth is accompanied by a transient inflammatory-like NF-κB activation in granulosa cells; our data place EDA-A2 at the apex of this response (Wang *et al*. 2002). The abrupt decline after the LH surge is consistent with progesterone-mediated NF-κB shutdown and prevents prolonged inflammation that could compromise oocyte quality (Yuan et al. 2018; Park et al. 2020). In contrast, sustained oviductal expression until 8 h post-hCG preserves an EDA-A2-rich condition precisely when ovulated COCs and newly ascending sperm meet in the ampulla. We therefore propose a two-step model: (1) ovarian EDA-A2 prepares the COC and follicular wall; (2) oviductal EDA-A2 continues to prime incoming sperm via EDA2R, ensuring that maximal fertilizing competence coincides with COC arrival. One limitation of this idea is that we have not yet directly detected the corresponding EDA-A2 protein in vivo. Therefore, validating the protein levels through immunostaining or western blotting is critical to strengthen the physiological relevance of our findings.

Fertilization is determined by a delicate balance of cytokines that regulate both sperm and oocytes at the moment of encounter. IL-6 released from cumulus cells stimulated by hyaluronidase activates JAK/STAT signaling in sperm, promoting tyrosine phosphorylation and excessive motility associated with the acquisition of fertilization capacity (Shimada *et al*. 2008; Lachance and Leclerc 2011). GM-CSF secreted from the human oviduct and endometrium provides supplementary nutritional support (Zhao *et al*. 1994; Giacomini *et al*. 1995). Adding this cytokine to embryo culture medium improves blastocyst formation rates and live birth rates (Adanacıoglu *et al*. 2022). On the other hand, pathological surges of TNF-α and IL-8 associated with infection reduce mitochondrial membrane potential, increase DNA fragmentation, inhibit sperm motility, and impair fertilization potential (Derbel *et al*. 2021). Our study identified EDA-A2, a ligand belonging to the TNF family, as an additional physiological regulatory factor. *Eda-a2* transcription in the ovaries and oviducts peaks just before ovulation and then decreases. This suggests that sperm encounter EDA-A2 at the time they reach the oviduct. Unlike TNF-α, EDA-A2 acts at low concentrations and does not cause oxidative damage. This suggests that EDA-A2 may constitute a beneficial option for the TNF superfamily, acting synergistically with IL-6/GM-CSF while inhibiting inflammatory TNF-α/IL-8 signaling. By targeting EDA alongside IL-6 and GM-CSF while strictly excluding TNF-α/IL-8, this approach provides a specific strategy to control the cytokine environment around fertilization and optimize assisted reproductive protocols.

TLR7 and TLR8, which are encoded by the X chromosome, are expressed in about half of the sperm, and that the agonist treatment may cause functional differences between X sperm and Y sperm (Umehara *et al*. 2019). EDA2R is not only encoded by gene localized on the X chromosome but also was expressed in the midpiece of mouse sperm. When sex determination was performed on embryos obtained through in vitro fertilization, there was a slight increase in XX embryos (60% of EDA vs. 50% of Ctrl; see Supplementary Fig. 3). This suggests that the EDA-A2/EDA2R pathway may be more easily activated in X sperm. However, since ligands are also expressed in vivo and the sex ratio in natural mating is 1:1, the use of EDA-A2 for sperm sexing is considered challenging. Nevertheless, at least from the results of this study, it is a fact that when sperm treated with EDA-A2 is used for in vitro fertilization, the fertilization rate is improved, and a large number of blastocyst embryos are obtained. The efficiency of in vitro fertilization greatly contributes to the efficient production of livestock and the successful of human infertility care. Due to interspecies differences in cytokine signaling and sperm physiology, claims regarding the translational relevance of the EDA-EDA2R axis remain hypothetical. Validation in domesticated species, and ultimately human germ cells is essential to determine whether the observed reproductive capacity enhancement effects are conserved across species.

In conclusion, we identify EDA-A2 as a fast-acting paracrine factor that links male capacitation dynamics to the female pre-ovulatory condition through its receptor EDA2R on sperm. This discovery broadens the functional repertoire of the EDA-A2 pathway beyond ectodermal morphogenesis and introduces a tractable molecular handle for improving reproductive success.

## Supplementary Information

Below is the link to the electronic supplementary material.

Supplementary file1 (DOCX 8.13 MB)

## Data Availability

Additional information needed to reanalyze the data reported in this paper is available from the primary contact upon request.
