# Supplementary material for: Functions of ectodysplasin A2 receptor (EDA2R) in inducing capacitation of sperm in mice
Source: In Vitro Cell Dev Biol Anim. 2025 Jul 21;61(8):1017–26. doi: 10.1007/s11626-025-01084-5 (PMC12589252; doi:10.1007/s11626-025-01084-5)

**Supplementary material**

**Supplemental Method**

**Embryo sexing by Polymerase Chain Reaction (PCR)**

The blastocysts obtained after in vitro fertilization were put individually into the tube containing 10 µl of 50 mM of NaOH and subjected to NaOH lysis at 100ºC for 20 minutes. Thereafter, PCR analysis was done with KOD FX Neo (TOYOBO Life Science, Osaka, Japan). RBM31 forward and reverse primers recognizing X and Y chromosome cells were used: 5′-CACCTTAAGAACAAGCCAATACA-3’ (forward) and 5′-GGCTTGTCCTGAAAACATTTGG-3’ (reverse). Electrophoresis was performed with PCR products using a 2% (w/v) agarose gel. Images were visualized in the chemiDoc imaging system.

**Supplemental Table 1**: HTF medium composition

| Components | HTF medium (/50 mL water) |
| --- | --- |
| NaCl | 297 mg |
| KCl | 17.5 mg |
| MgSO4・7H2O | 2.5 mg |
| KH2PO4 | 2.5 mg |
| glucose | 25.1 mg |
| Na-lactate | 174.5 µL |
| CaCl2・2H2O | 14.9 mg |
| NaHCO3 | 105 mg |
| Na-pirvate | 1.8 mg |
| Bobine Serum Albumin | 250 mg |

It was supplemented with antibiotics: 100 units/mL of penicillin G and 100 μg/mL of streptomycin.

**Supplemental Figures and Figure legends**

**Supplemental Figure 1. Gating strategy of flow cytometry**

(A) Gating strategy for the selection of single sperm. Using forward scatter (FSC)-A and side scatter (SSC)-A dot plots, cells of similar size and complexity were first selected (R1). (B) Representative images of single sperm in the R1 gate captured using the Attune CytPix Flow Cytometer. (C) The dot plots of fluorescein isothiocyanate-conjugated peanut agglutinin (PNA-FITC; x-axis) and propidium iodide (PI; y-axis). Sperm was stained with a single dye after being permeabilized with phosphate buffer containing 0.3% Triton. The threshold was set to ensure that more than 95% of each signal was positive. Un-stained sperm was used as negative control.

**Supplemental Figure 2. Effects of EDA-A2 supplementation on mouse sperm motility**

Box plots represent velocity parameters of epididymal sperm incubated with different concentrations of EDA-A2 ligand for 60 min. VAP; average path velocity, VSL; Straight-Line Velocity, VCL; Curvi-Linear Velocity, ALH; amplitude of lateral head displacement.

**Supplemental Figure 3. The treatment with EDA-A2 ligand enabled sex preselection for IVF**

(A) The sexing of blastocyst embryos derived from IVF using the sperm treated with EDA-A2. Left image: sex ratios of embryos using the sperm incubated in HTF medium (Ctrl). Middle image: sex ratios of embryos using the sperm preincubated in HTF medium contained 1000 ng/mL EDA-A2 for 60 min (EDA). Upper band denotes the presence of the Y chromosome, and lower band denotes the presence of the X chromosome. (B) Ratio of XX embryos or XY embryos in IVF using the sperm treated with EDA-A2. Values are the mean ± SEM of three replicates.

**Supplemental Figure 1**


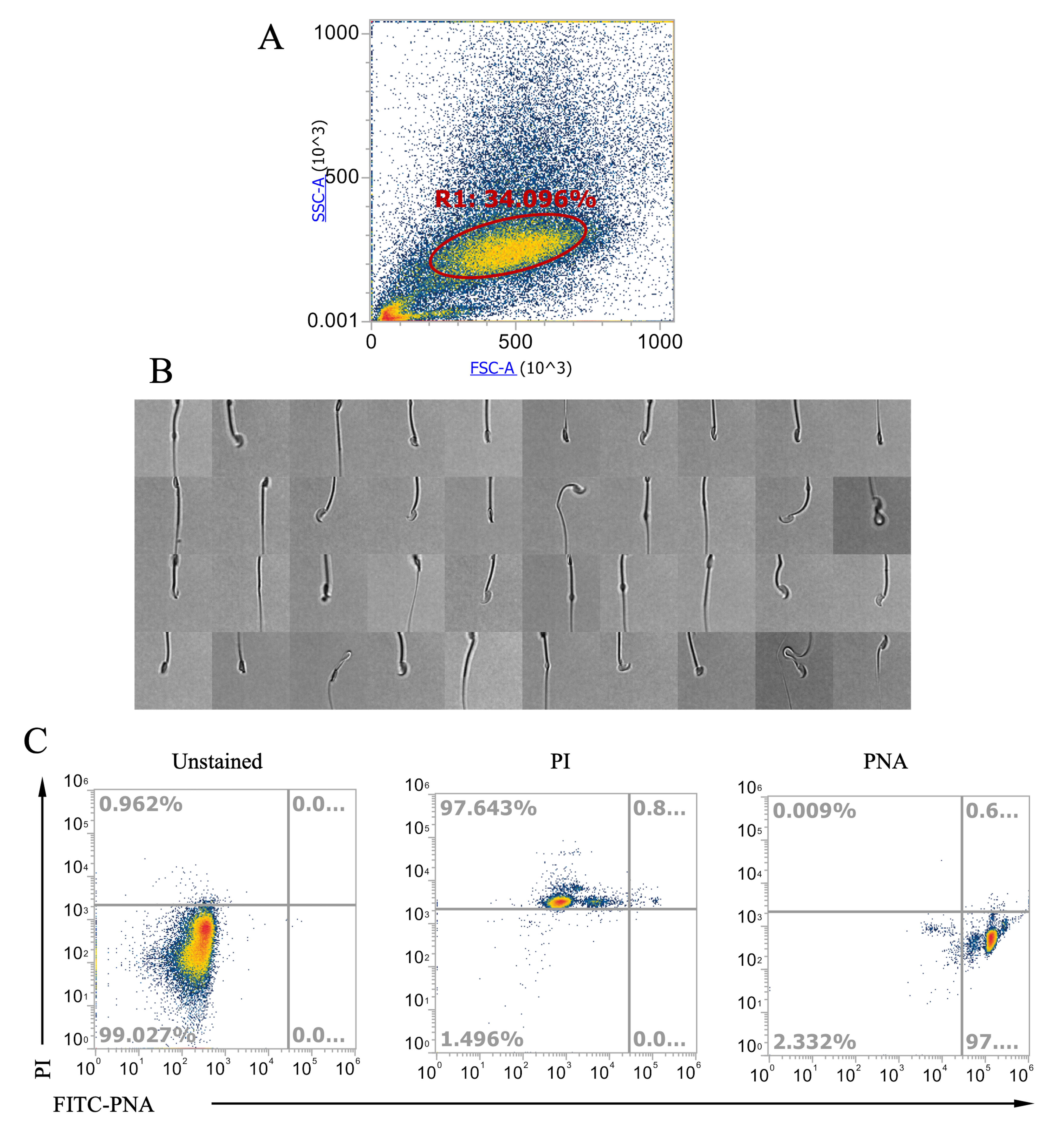


**Supplemental Figure 2**


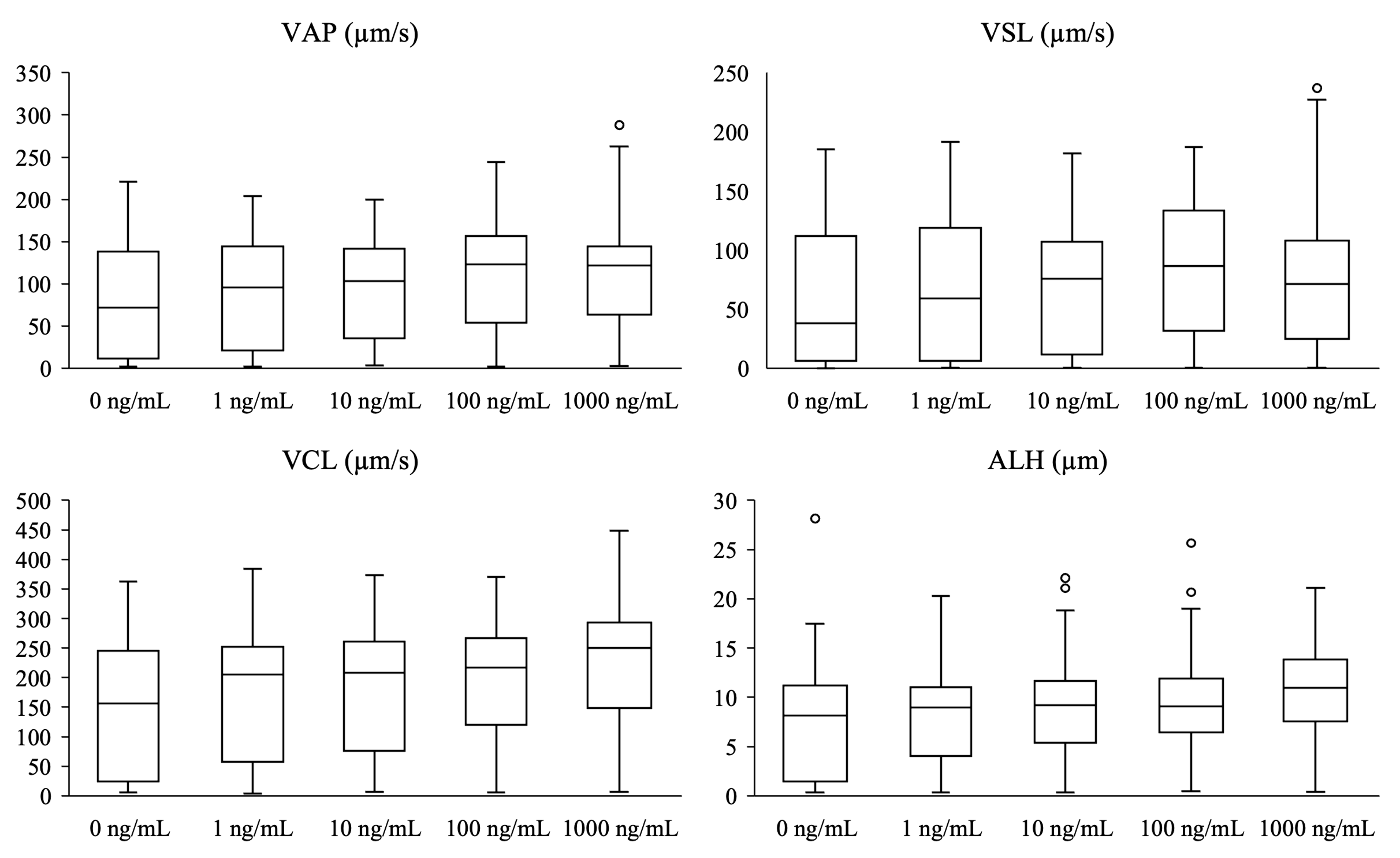


**Supplemental Figure 3**


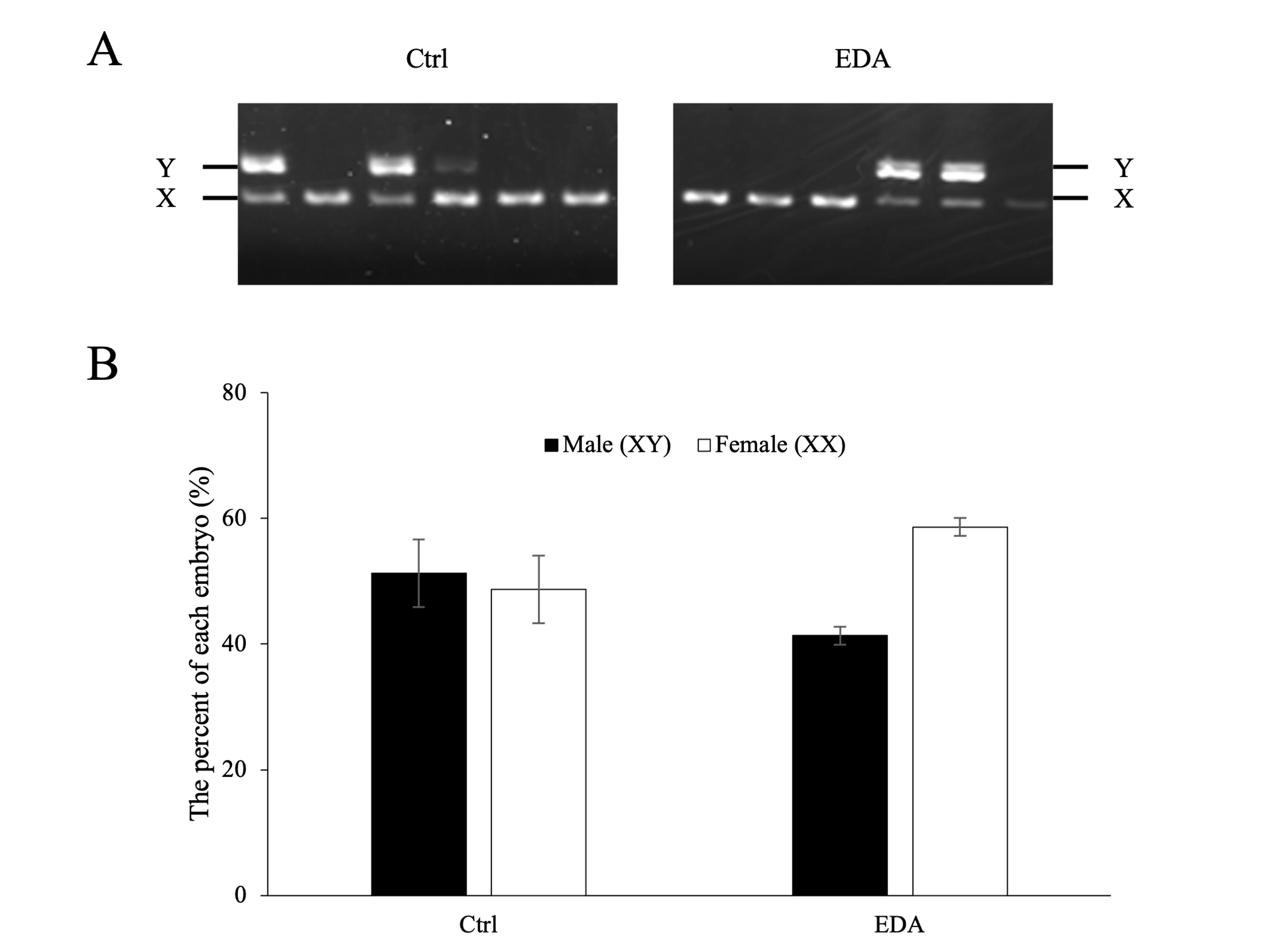

Supplement: Supplementary file 1 — Supplementary file1 (DOCX 8.13 MB) [file 11626_2025_1084_MOESM1_ESM.docx]
